# Aquatic physiotherapy: a vestibular rehabilitation option

**DOI:** 10.1016/j.bjorl.2019.12.003

**Published:** 2020-01-13

**Authors:** Carolina Maria Maia Pereira, Jalene de Sarah Pinheiro do Vale, Wellington Pinheiro de Oliveira, Denise da Silva Pinto, Renato Valeiro Rodrigues Cal, Yaná Jinkings de Azevedo, Fayez Bahmad

**Affiliations:** a Universidade de Brasília (UnB), Faculdade de Ciências da Saúde (FCS), Programa de Pós-Graduação (PPG), Brasília, DF, Brazil; b Centro Universitário do Estado do Pará (CESUPA), Belém, PA, Brazil; c Universidade Federal do Pará (UFPA), Faculdade de Fisioterapia e Terapia Ocupacional, Belém, PA, Brazil; d Universidade Federal do Pará (UFPA), Belém, PA, Brazil; e Hospital Universitário de Brasília, Brasília, DF, Brazil; f Universidade de Brasília (UNB), Faculdade de Ciências de Saúde - Asa Norte, Programa de Pós-Graduação em Ciências da Saúde, Brasília, DF, Brazil

**Keywords:** Vestibular system, Vestibular rehabilitation, Aquatic physiotherapy, Dizziness

## Abstract

**Introduction:**

Vestibular rehabilitation is a fundamental resource for vestibular symptom control. Its performance in the aquatic environment is considered safe for the physical activities of the elderly, because they act simultaneously on musculoskeletal disorders and balance improvement.

**Objective:**

To evaluate the effects of an aquatic physiotherapy protocol in individuals with peripheral vestibular alterations.

**Methods:**

This was an interventional case study with a paired intentional sample of four subjects, who were selected for convenience. The subjects, all of them diagnosed with peripheral vestibulopathy, were submitted to twelve sessions of aquatic physiotherapy for vestibular rehabilitation, being evaluated for dizziness in three moments: initial, after six sessions and at the end of the sessions. The tests applied were: unipodal support to measure static balance, the Fukuda stepping test, which estimates the dynamic balance and the dizziness handicap inventory protocol, aimed at verifying how dizziness influences daily life.

**Results:**

When analyzing the static balance, initially the individuals were in the adaptive and abnormal dimensions, and all reached normality at the end of the protocol. Regarding the dynamic balance, the individuals initially showed marked impairment in the angular deviation, mainly to the side of pathology (75% to the left and 25% to the right), achieving improvement at the end of the study. However, it failed to reach statistical significance. The dizziness handicap inventory showed a statistically significant difference in its totality (*p* = 0.0414), which addresses the physical, functional and emotional factors.

**Conclusion:**

In conclusion, the aquatic physiotherapy protocol for vestibular rehabilitation of patients with peripheral impairment was positively assessed by the participants, considering the improvement in dizziness (static and dynamic) and its impact on daily activities.

## Introduction

Body balance is the result of motor skills experienced since childhood, when transfers such as rolling, crawling, standing, and finally walking start. The harmonic performance of this process is due to the integration of three systems: the visual system to maintain the stabilization of body sway coordinated by the cerebellum; the proprioceptive system maintained by the muscle spindles; and the vestibular system, purely exproprioceptive (corresponds to the sensation of position and movement of a body part in relation to the environment), consisting of a set of organs located in the inner ear, able to detect body movements and thus to maintain balance.^1^ These three systems work in harmony, governed by the central nervous system, which in turn produces reflexes (vestibulo-ocular, vestibulo-spinal and vestibulo-cervical), which are automatically and unconsciously generated, ensuring the postural balance necessary for interaction with the environment, whether at rest (static equilibrium) or in movement (dynamic equilibrium).^2^, ^3^

The equilibrium sensations are detected by the vestibular system, which is divided into the Central Vestibular System (CVS), consisting of the vestibular nuclei and the brainstem vestibular pathways, and the Peripheral Vestibular System (PVS), consisting of the bony (cochlea and vestibule) and the membranous labyrinth (otolithic organs – saccule and utricle; and three semicircular canals), located within the temporal bone on each side of the skull. The semicircular canals have receptors for angular accelerations and saccular and utricular macula have receptors for linear accelerations and gravity action.^3^, ^4^, ^5^

For the prevention of falls, it is important to improve the sensory information receptors of the vestibular, visual and somatosensory system, which activate antigravity muscles and stimulate balance. Approximately 30% of people over 65 are known to fall at least once a year and 15% at least twice a year, and this risk increases with age. These falls can result in several morbidities for an individual, leading to lack of independence, and reduced quality of life.^6^ One of the treatments that provides this stimulation is the practice of physical activity. Therefore, the use of hydrotherapy is found in the literature as a way to treat several diseases caused by aging (rheumatic, neurological and orthopedic ones). However, only recently have scientific researches emerged to show its use in other areas. The aquatic environment is considered safe for the elderly due to the physical properties of water – relative density, fluctuation, viscosity, surface tension, hydrostatic pressure and turbulence, and when added to physical exercise, it acts simultaneously on musculoskeletal disorders and balance improvement.^7^, ^8^

The implementation of a physiotherapy protocol to assist the population with peripheral vestibular system pathologies that cause balance disorders, together with the aquatic environment, may be beneficial, considering the risk of falls. The combination of the characteristics of aquatic physiotherapy and vestibular rehabilitation exercises described in the literature has shown positive results in vestibular compensation due to the neuroplasticity capacity of this system.^9^ Thus, repeated head movements and eye stabilization can be performed with greater confidence in the aquatic environment due to the latter’s properties.^9^ Moreover, vestibular rehabilitation therapy has shown to be effective, reliable and noninvasive, which has led to considering it an important treatment tool.^10^ According to these observations, this study proposes aquatic physiotherapy as an option for vestibular rehabilitation.

## Methods

This is an intervention study, carried out in a paired sample performed at the Aquatic Physiotherapy Service of the Physiotherapy School Clinic, over a period of one month, with a frequency of three times a week, totaling 12 sessions lasting 50 min. It was approved by the Research Ethics Committee (CEP CAAE: 4810.0.000.323-09).

The inclusion criteria for the study comprised individuals diagnosed with Peripheral Vestibular Deficit complaining of dizziness and of static and dynamic balance alterations and show safety and independence for the activities in the water. Patients with central vestibular alterations, those using gait aids, hearing and visual impairment, as well as contraindications for aquatic physiotherapy were excluded from the study.

The present study consisted of an intentional sample of four individuals selected for convenience, among patients followed at the Outpatient Otorhinolaryngology Service. All individuals signed the Free and Informed Consent Form.

### Procedures

The intervention started after the selected individuals completed a physiotherapy evaluation form containing personal information; a brief history of the clinical picture; blood pressure, heart rate, respiratory rate, weight and height measurements; collection of dizziness characteristics; static (Unipodal Support Test) and dynamic (Fukuda Stepping Test) balance tests were applied before the intervention, after six sessions, and at the end, after the twelve sessions. The abovementioned tests are described below.

Unipodal Support Test: the patient stabilized gaze at one point and then removed one leg from the floor, standing only on the other leg for a maximum of 30 seconds, repeating the test with the other side. The classification was given at three levels, with scores between 2 and 6 points: Normal (3 points), where the individual is able to maintain unipodal support for 30 seconds without any other assistance; Adaptive (2 points), where the individual is able to maintain unipodal support for only 15 seconds without support; Abnormal (1 point), characterized by the inability to maintain unipodal support or for less than 15 seconds.^11^

The Fukuda Stepping Test is performed on three concentric circles drawn on the floor, of which radii are 0.5 m apart. These circles are divided into 12 equal parts by straight lines crossing the center at an angle of 30°. The patient is placed in the center of the smallest circle and asked to march, raising the knees at approximately 45° without moving, performing 60 steps (one per second), with outstretched arms and closed eyes. After one minute, pathological results are considered when there is displacement greater than 0.5 m and/or rotation greater than 30°. This test is useful in monitoring patients with peripheral pathologies during treatment, as it provides signs of vestibular compensation.^12^

After filling out the physiotherapy evaluation form, a questionnaire was applied to assess the difficulties experienced by patients regarding dizziness or imbalance, called the Dizziness Handicap Inventory (DHI) table, applied before the protocol and after the 12 sessions. The purpose of this scale is to identify the difficulties the subject may be experiencing due to dizziness or imbalance. After answering the 25 questions, divided into subgroups of Functional (F), Emotional (E) and Physical (P) components, this survey provides a quantification of how the patient perceives their imbalance and its impact on activities of daily life, being useful to determine subjective improvement. Each “yes” answer corresponds to 4 points; “sometimes” gets 2 points; and “no” gets 0 points.^13^

### Intervention

The Aquatic Vestibular Rehabilitation protocol was adapted based on the unification of the following protocols: Aquatic Physiotherapy for Vestibular Rehabilitation (FARV, *Fisioterapia Aquática para Reabilitação Vestibular*), based on the physical effects of water on the immersed body, Halliwick and Bad Ragaz exercises for aquatic physiotherapy, together with the *Associazone Otologi Ospedalieri Italiani* method for vestibular rehabilitation on the floor;^14^ the adaptation and water balance improvement exercises;^15^ and the Cawthorne-Cooksey Exercises, which describe the vestibular rehabilitation (VR) protocols developed on the floor.^16^, ^17^ The following principles were taken into account to adapt the exposed protocols: (i) The adaptation of patients to the aquatic environment; (ii) The complexity and comprehensiveness of the exercises; (iii) Repetitive head movements; and (iv) Postural and gait transfers associated with sensory deprivation and/or conflict.

The periodization of the aquatic vestibular rehabilitation protocol was adapted to four weeks, with three weekly sessions of 50 min totaling 12 sessions, which were performed in group, with pool temperature of around 32 °C and the water depth up to the individuals’ xiphoid process.

The aquatic physiotherapy session was divided into three moments: the first was characterized by the patient’s adaptation to the aquatic environment, consisting of six exercises lasting 2 min on average, totaling 12 min; the second consisting of Cawthorne–Cooksey exercises adapted for water, dislocation, postural transfer and rotational trunk control exercises, which lasted an average of 2 min each, totaling 26 min; and at the third moment, a playful activity and stretching lasting 5 min each, totaling 50 min of activity, was developed.

As the individuals performed the proposed exercises with more dexterity, the degree of difficulty also increased. Therefore, in the first week, ten repetitions of each exercise were performed with the performance velocity controlled by the patients themselves, while in the second week, fifteen repetitions of each movement were required, while maintaining the velocity. The same series was maintained in the third week, but at a more intense velocity, and in the last week the movements were performed with the first ten repetitions with the eyes open and the last ten with the eyes closed. The description of the exercises is shown in Appendix A of the supplementary document, as well as the evaluation sheet and other documents of this article.

### Data analysis

According to the nature of the variables, a descriptive statistical analysis was performed, generating a database, as well as tables and charts. To analyze the significance of the obtained results, the initial, intermediate and final comparison of the effects of the study intervention were carried out using Friedman’s test for the unipodal support; the ANOVA test for the Fukuda test, and the Student's *t* test for the survey of dizziness deficiency (DHI), considering a level α = 0.05. These analyses were performed using the BioEstat 5.0 software.

## Results

Four individuals diagnosed with right and left vestibular neuritis and benign vertigo of aging were included in the sample. The studied age range ranged from 54 to 75 years, including both genders, being 75% females and 25% males. All had complaints of dizziness and alteration of static and dynamic balance. Table 1 shows the description of the present study sample. The most common pathology identified in the patients was vestibular neuronitis.

**Table 1:** Description of the individuals involved in the study (n = 4), considering the data obtained from the anamnesis of the aquatic physiotherapy protocol.

Patients	Clinical diagnosis	Age	Gender	First crisis	Medication
MHGE	Left vestibular neuronitis	57 years	F	2010	No
SSP	Left benign vertigo of the aging	75 years	F	1995	No
VRL	Right vestibular neuronitis	54 years	F	2005	No
STS	Left vestibular neuronitis	65 years	M	2008	No

The patients reported the onset of the first crisis between nine and twenty-four years before. It was also observed that none of the patients used specific medication to treat dizziness; therefore, with no interference regarding the effects of the physiotherapy treatment protocol.

All patients had a sudden onset of the dizziness complaint, described in Table 2, which also shows the characteristics of each individual’s dizziness according to its intensity, occurrence and duration.

**Table 2:** Description of the individuals involved in the study (n = 4) according to the characteristics of dizziness in the initial phase of the aquatic physiotherapy protocol application.

Patients	Onset	Intensity	Occurrence	Duration
MHGE	Sudden	Intense	Very frequent	Days
SSP	Sudden	Moderate	Sporadic	Seconds
VRL	Sudden	Variable	Sporadic	Days
STS	Sudden	Intense	Frequent	Minutes

The dependent variables considered in the study were the static balance, the dynamic balance, the impairment perceived by the patient and the impact of dizziness on activities of daily life, shown in Table 3, Table 4, Table 5. In the analysis of the results regarding the static balance of the individuals in the present study, the following mean and standard deviation (SD) values were obtained at the initial (3.7 ± 1.5), intermediate (5.2 ± 0.9) and final (6 ± 0) evaluations. Therefore, we observed that initially, the individuals were in the adaptive and abnormal dimensions (values < 6), and in the end all of them reached normality (value = 6), confirmed by an SD = 0. The *p* value found for this analysis was 0.1054, showing no statistically significant difference at the Friedman’s test, considering an α ≤ 0.05 (5%), as shown in Table 3.

**Table 3:** Static balance evaluation through the Unipodal Test of patients involved in the study (n = 4) in the initial, intermediate and final phase of the aquatic physiotherapy protocol application.

Parameters	Unipodal Test		
	Initial	Intermediate	Final
Minimum	2	4	6
Maximum	5	6	6
Median	4	5,5	6
Mean	3.7	5,2	6
SD	1.5	0,9	‒
*p*-value^a^	0.1054		

SD, Standard Deviation; (‒), Numeric data equal to zero, not resulting from rounding.

a Friedman’s test.

**Table 4:** Dynamic balance evaluation of the patients involved in the study (n = 4) using the Fukuda stepping test in the initial, intermediate and final phase of the aquatic physiotherapy protocol application.

Parameters	Angular deviation			Distance from the starting point		
	Initial	Intermed.	Final	Initial	Intermed.	Final
Minimum	60	‒	‒	1	‒	0,5
Maximum	120	90	180	1,5	1,5	1
Median	75	30	30	1,2	1,2	0,7
Mean	82,5	37,5	60	1,2	1	0,7
SD	28,7	37,7	81,2	0,3	0,7	0,3
*p*-value^a^	0,5313			0,3676		

SD, Standard Deviation; (‒), Numeric data equal to zero, not resulting from rounding.

a 1-way ANOVA test.

**Table 5:** Evaluation of the physical, functional and emotional aspects of the patients involved in the study (n = 4) before and after the aquatic physiotherapy protocol application.

Patient	Physical		Functional		Emotional		Total	
	Initial	Final	Initial	Final	Initial	Final	Initial	Final
MHGE	28	‒	32	‒	40	‒	100	‒
SSP	18	‒	8	‒	8	‒	34	‒
VRL	16	‒	14	‒	8	‒	38	‒
STS	26	16	32	18	36	18	94	52
Mean	22	4	21.5	4.5	23	4,5	66,5	13
SD	5.9	8.0	12.4	9.0	17,4	9,0	35,3	26,0
*p*-value	0.0171^a^		0.0467^a^		0.0914		0.0414^a^	

SD, Standard Deviation; (‒), Numeric data equal to zero, not resulting from rounding.

a Student’s *t* test for paired samples.

In Table 4, the dynamic balance was measured in the study subjects using the Fukuda stepping test through the angular deviation dimension, obtaining the following values in the initial, intermediate and final evaluations: 82.5 ± 28.7; 37.5 ± 37.7 and 60 ± 81.2. In the dimension of distance from the starting point, the following values were obtained respectively in the initial, intermediate and final evaluations: 1.2 ± 0.3, 1 ± 0.7 and 0.7 ± 0.3. The degrees identified in the angular deviation were formed by the rotational displacement from the starting point to the stopping point, always deviating to the side affected by the pathology, tending to 75% to the left and 25% to the right.

Table 5 describes the values obtained in the assessment of the impairment due to dizziness (DHI), where the following values were obtained respectively for the physical dimension at the initial and final evaluations: 22 ± 5.9 and 4 ± 8; with *p* = 0.0171. In the functional dimension, the following values were obtained respectively in the initial and final evaluations: 21.5 ± 12.4 and 4.5 ± 9; with *p* = 0.0467, both of which showed a statistically significant difference. In the emotional dimension, the following values were obtained in the initial and final evaluations: 23 ± 17.4 and 4.5 ± 9; with *p* = 0.0914, showing no statistically significant difference. Adding all the dimensions, the following values were obtained in the initial and final evaluations: 66.5 ± 35.3 and 13 ± 26; with *p* = 0.0414, showing a statistically significant difference, with a value of α = 0.05 (5%) for all dimensions.

## Discussion

According to some studies, the prevalence of neurotological dysfunctions, such as vestibular neuronitis, is associated with advancing age and the female gender, with an incidence of 76.6%, a fact observed in the present study in which 75% of the affected individuals were females.^17^

In a study that evaluated chronic vestibular disorder in the elderly with complaints of dizziness and/or imbalance with more than three months of disease onset, the authors reported that individuals with dizziness that occurred everyday had worse balance performance when compared to those who reported sporadic dizziness, probably due to the greater limitation imposed by the dizziness, which was more frequent.^18^ This event was also evidenced in the present study because individuals with frequent or very frequent (daily) dizziness events had worse performance compared to those with sporadic occurrence.

Another study evaluated the postural balance of a group of elderly individuals who practiced hydrotherapy (n = 11). At their evaluation of the initial static balance, they obtained a mean of 35.55 ± 2.01. After 10 therapeutic intervention sessions, a statistically significant improvement was obtained, with a mean of 38 ± 0.89 and *p* = 0.002.^19^ Comparing it with the present study, it can be stated that regarding the mean of the initial (3.7 ± 1.5) and final (6 ± 0) evaluations, both were inferior to those of the related studies, which can be justified by the fact that the sample sizes were different.

Researchers evaluated the static balance in elderly individuals after 72 exercise sessions compared with the control group, obtaining a mean of 15.56, with a *p* value = 0.046.^20^ The current study compared the mean before and after the intervention in the same group, resulting in a mean of 4.9 and a *p* value = 0.1054; observing that although the mean is lower than that of the comparative study, the result did not reach statistical significance, and such differences may be justified by the number of sessions, leading to a longer follow-up.

Analyzing another study that compares the dynamic balance, it obtained a mean of 11.33 at the end of the intervention, with a value of *p* = 0.029.^21^ The present study showed a mean of 60 and 0.7 and a value of *p* = 0.5313 and *p* = 0.3676, reaching higher values than the mean and the *p* value did not reach statistical significance.

In another study, they applied VR exercises in a group of 10 elderly individuals with chronic peripheral vestibular disorders for 12–16 sessions. They evaluated the dynamic balance before and after the intervention, reaching a value of *p* = 0.005 (total).^22^ The present study shows a *p* value = 0.5313 and *p* = 0.3676 for the dynamic balance tests, considering that Table 4 shows an improvement in this dimension, although it does not reach statistical significance.

Studies state that dizziness is the most common complaint after the age of 65, favored by multiple factors associated with aging, such as balance deficit, postural hypotension and vestibular dysfunction itself.^3^ This leads us to believe that individuals with Fukuda Stepping test sensitivity need long-term treatment, as the study subjects are on average 70 years old.

In studies in which the Student's *t* test was also used for paired samples, it showed a statistically significant difference in total for DHI (*p* = 0.01).^23^ This is in agreement with the results obtained in the current study, showing a statistically significant difference in the total aspect of DHI (*p* = 0.0414).

The study of a group of eight elderly individuals with dizziness complaints who were submitted to the Cawthorme and Cooksey Exercises for 120 sessions resulted in a total average of 6.50 ± 7.69, in which the three dimensions, physical (0.00413), functional (0.00006) and emotional (0.03268), reached statistical significance with *p* = 0.00081 (total).^24^ In the current study, the physical (0.0171) and functional (0.0467) dimensions reached statistical significance, but the emotional dimension (0.0914) was not satisfactory, however at a *p* value = 0.0414 (total) it was statistically significant.

Although the study did not reach statistical significance regarding the static and dynamic balance, the subjects showed improvement at the end of the aquatic physiotherapy protocol application. According to the analyses, even though the majority of the individuals showed improvement regarding their dizziness complaints with VR, each individual responded differently to the therapy, due to several aspects such as psychoadaptation to the pathology, intensity and frequency of dizziness, among others. Moreover, the water assists in the fall response, promoting a better body balance reaction.^14^

When the static and dynamic balance of patients with peripheral vestibular alterations was taken into consideration and after the final evaluation, an improvement was observed. However, due to the fact that an individual in the sample was affected by respiratory disease, interrupting the protocol for one week and then returning, the standard deviation failed to reach a significance level.

Regarding the deficiency perceived by the patient himself and the impact of dizziness on daily activities, assessed by the DHI, we observed an improvement in the dizziness complaints of all patients, especially in the physical and functional dimensions.

The aquatic physical therapy protocol aimed at vestibular rehabilitation of patients with peripheral impairment was positively evaluated by the participants, considering the improvement in dizziness (static and dynamic) and its impact on daily activities.

## Conclusion

It was concluded that the aquatic physiotherapy protocol for vestibular rehabilitation in patients with peripheral vestibular diseases showed to be efficient and easy to apply. Therefore, we observed that the aquatic environment favored greater confidence and safety for patients to perform their vestibular recovery and resulted in an improvement in the occurrence, intensity and duration of dizziness complaints.

## Conflicts of interest

The authors declare no conflicts of interest.
